# More efficient approaches to the exponentiated half-logistic distribution based on record values

**DOI:** 10.1186/s40064-016-3047-y

**Published:** 2016-08-30

**Authors:** Jung-In Seo, Suk-Bok Kang

**Affiliations:** 1 Department of Statistics, Daejeon University, 62, Daehak-ro, Dong-gu, Korea; 2 Department of Statistics, Yeungnam University, 280, Daehak-ro, Gyeongsan, Korea

**Keywords:** Exponentiated half-logistic distribution, Hierarchical Bayesian model, Record value, Pivotal quantity, Robust estmation

## Abstract

The exponentiated half-logistic distribution has various shapes depending on its shape parameter. Therefore, this paper proposes more efficient approach methods for estimating shape parameters in the presence of a nuisance parameter, that is, a scale parameter, from Bayesian and non-Bayesian perspectives if record values have an exponentiated half-logistic distribution. In the frequentist approach, estimation methods based on pivotal quantities are proposed which require no complex computation unlike the maximum likelihood method. In the Bayesian approach, a robust estimation method is developed by constructing a hierarchical structure of the parameter of interest. In addition, two approaches address how the nuisance parameter can be dealt with and verifies that the proposed methods are more efficient than existing methods through Monte Carlo simulations and analyses based on real data.

## Background

Record values introduced by Chandler (1952) arise in many real-world situations involving weather, sports, economics, life-tests and stock markets, among others. Let $\{X_1, \, X_2, \, \ldots \}$ be a sequence of independent and identically distributed (iid) random variables (RVs) with a cumulative distribution function (CDF) and a probability density function (PDF). Lower records are values in the sequence lower than all preceding ones, and the observation $X_1$ is the first record value. Indices for which lower record values occur are given by record times $\{L(k), \, k\ge 1\}$, where $L(k)=\min \{j|j>L(k-1), \, X_{j}<X_{L(k-1)}\}, \, k>1$, with $L(1)=1$. Therefore, a sequence of lower record values is denoted by $\{X_{L(k)}, k=1,2,\ldots \}$ from the original sequence $\{X_1, \, X_2, \ldots \}$. However, because record occurrences are rare in practice, the maximum likelihood method can entail a substantial bias for inferences based on record values. Alternately, a method based on a pivotal quantity can be considered. Some authors studied estimation methods based on pivotal quantities when censored samples or record values are observed. Wang and Jones (2010) proposed an estimation method based on a pivotal quantity if progressively Type-II censored samples are observed from a certain family of two-parameter lifetime distributions. Yu et al. (2013) provided new estimation equations using a pivotal quantity and showed that those equations to be particularly effective for skewed distributions with small sample sizes and censored samples. Wang et al. (2015) constructed confidence and predictive intervals by using some pivotal quantities for a family of proportional reversed hazard distributions based on lower record values. Seo and Kang (2015) provided an estimation equation more efficient than the maximum likelihood equation for the half-logistic distribution (HLD) based on progressively Type-II censored samples. As another alternative, the Bayesian approach can be effective if sufficient prior information can be obtained. Madi and Raqab (2007) assumed that unknown parameters of the two-parameter exponentiated exponential distribution (EED) have independently distributed gamma priors and predicted the subsequent record values based on observed record values. Asgharzadeh et al. (2016) developed estimation methods for obtaining the maximum likelihood estimators (MLEs) and the Bayes estimators of the unknown parameters in the logistic distribution based on the upper record values, and suggested the use of the Bayesian method if there is reliable prior information. However, because their approach is based on a subjective prior, it can lead to incorrect estimation results if there is no sufficient prior information. In this case, two alternatives can be considered: estimation based on noninformative or objective priors and that, based on a hierarchical prior obtained by mixing hyperparameters of a natural conjugate prior. In this regard, Jeffreys (1961) and Bernardo (1979) introduced the Jeffreys prior and a reference prior, respectively. Xu and Tang (2010) derived a reference prior for unknown parameters of the Birnbaum-Saunders distribution. Fu et al. (2012) developed an objective Bayesian analysis method to estimate unknown parameters of the Pareto distribution based on progressively Type-II censored samples. Kang et al. (2014) developed noninformative priors for the generalized half-normal distribution when scale and shape parameters are of interest, respectively. Seo et al. (2014) provided a hierarchical model of a two-parameter distribution with a bathtub shape based on progressively Type-II censoring, which leads to robust Bayes estimators of unknown parameters of this distribution. Seo and Kim (2015) developed Bayesian procedures to approximate a posterior distribution of a parameter of interest in models with nuisance parameters and examined the sensitivity of the posterior distribution of interest in terms of an information measure for the Kullback–Leibler divergence.

This paper proposes more efficient methods for estimating unknown parameters of the exponentiated HLD (EHLD) based on lower record values by employing these alternatives from Bayesian and non-Bayesian perspectives. In the frequentist approach, the proposed estimation methods require no complex computation unlike the maximum likelihood method. In the Bayesian approach, a robust estimation method is developed by constructing a hierarchical structure of the parameter of interest. In addition, two approaches address how the nuisance parameter can be dealt with. The efficiency is proved through Monte Carlo simulations and analyses based on real data in “Application” section. The CDF and PDF of the EHLD are respectively given by

$$\begin{aligned} F(x)=\left( \frac{1-e^{-\theta x}}{1+e^{-\theta x}} \right) ^\lambda \end{aligned}$$

and

$$\begin{aligned} f(x)=\theta \lambda \left( \frac{1-e^{-\theta x}}{1+e^{-\theta x}} \right) ^\lambda \frac{2e^{-\theta x}}{1-e^{-2\theta x}}, \quad x>0, \; \theta , \, \lambda >0, \end{aligned}$$

where $\theta$ is the reciprocal of a scale parameter and $\lambda$ is the shape parameter. Recently, this distribution has received considerable attention as a generalized distribution of the HLD. Seo and Kang (2014) derived the entropy of the another generalized version of the HLD for Type-II censored samples observed from the distribution. Seo and Kang (2014) provided Bayesian estimation and prediction methods for the EHLD based on record values. Seo and Kang (2015) proved that the EHLD can be an alternative to the gamma distribution or the EED with two parameters (scale and shape). In addition, they showed that the PDF of the EHLD is a decreasing function for $\lambda \le 1$ and a right-skewed unimodal function for $\lambda >1$. These findings indicate that the EHLD based on record values is a skewed distribution with small samples for $\lambda >1$. In this case, the maximum likelihood method may not appropriate because it is useful for large samples. Therefore, this paper provides estimation methods more efficient than the maximum likelihood method in terms of the mean squared error (MSE) and bias as well as the computational cost in estimating a parameter of interest in a presence of the nuisance parameter and proposes a robust Bayesian estimation method using the hierarchical structure of a prior distribution. Here the paper focuses on estimating the shape parameter $\lambda$ because the EHLD has various shapes depending on its shape parameter and the scale parameter is a nuisance parameter.

The rest of this paper is organized as follows: “Frequentist estimation” section proposes estimation methods based on pivotal quantities that require no complex computation and are more efficient than the maximum likelihood method in terms of the MSE and bias if lower record values arise from the EHLD. “Bayesian Estimation” derives a reference prior for unknown parameters, and then proposes a robust Bayesian estimation method by constructing a hierarchical structure of the parameter of interest of the EHLD based on lower record values. “Application” compares numerical results for the MSE and bias and analyzes real data, and “Conclusion” concludes the paper.

## Frequentist estimation

This section estimates the parameter of interest $\lambda$ in the presence of a nuisance parameter $\theta$. MLEs and corresponding approximate confidence intervals (CIs) are derived, and then estimation methods are proposed based on pivotal quantities that not only are more convenient to compute but also can provide better results in terms of the MSE and bias.

### Maximum likelihood estimation

Let $X_{L(1)}, \ldots , X_{L(k)}$ be the first *k* lower record values from the EHLD. Then by the definition of Arnold et al. (1998), the likelihood function based on these lower record values is given by

1 $$\begin{aligned} L(\lambda , \theta )&=f(x_{L(k)})\prod _{i=1}^{k-1}\frac{f(x_{L(i)})}{F(x_{L(i)})} \nonumber \\&=\theta ^k \lambda ^k \left( \frac{1-e^{-\theta x_{L(k)}}}{1+e^{-\theta x_{L(k)}}} \right) ^\lambda \prod _{i=1}^{k} \frac{2e^{-\theta x_{L(i)}}}{1-e^{-2\theta x_{L(i)}}}. \end{aligned}$$

The log-likelihood function is given by

2 $$\begin{aligned} \log L(\lambda , \theta )\,=\,&k \log \theta + k \log \lambda + k \log 2 + \lambda \log \left( \frac{1-e^{-\theta x_{L(k)}}}{1+e^{-\theta x_{L(k)}}}\right) -\theta \sum _{i=1}^k x_{L(i)} \nonumber \\&- \sum _{i=1}^k \log \left( 1-e^{-2 \theta x_{L(i)} }\right) . \end{aligned}$$

From (2), the likelihood equations for $\lambda$ and $\theta$ are given respectively by

3 $$\begin{aligned} \frac{\partial }{\partial \lambda } \log L(\theta , \lambda )&= \frac{k}{\lambda }-\log \left( \frac{1+e^{-\theta x_{L(k)}}}{1-e^{-\theta x_{L(k)}}}\right) \nonumber \\&=0 \end{aligned}$$

and

4 $$\begin{aligned} \frac{\partial }{\partial \theta } \log L(\theta , \lambda )&=\frac{k}{\theta }+\lambda \frac{2e^{-\theta x_{L(k)}}}{1-e^{-2\theta x_{L(k)}}} x_{L(k)} - \sum _{i=1}^{k} \frac{1+e^{-2\theta x_{L(i)}}}{1-e^{-2\theta x_{L(i)}}} x_{L(i)} \nonumber \\&=0. \end{aligned}$$

Then the MLE of $\lambda$ can be easily obtained for known $\theta$, by solving the likelihood equation (3) for $\lambda$ as follows:

$$\begin{aligned} \hat{\lambda }(\theta ) =\frac{k}{h_1(\theta )}, \end{aligned}$$

where

$$\begin{aligned} h_1(\theta )=\log \left( \frac{1+e^{-\theta x_{L(k)}}}{1-e^{-\theta x_{L(k)}}}\right) . \end{aligned}$$

If both $\lambda$ and $\theta$ are unknown, then MLEs $\hat{\theta }$ and $\hat{\lambda }$ can be obtained simultaneously by solving Eqs. (3) and (4) through the Newton–Raphson method. An asymptotic variance-covariance matrix of MLEs can be obtained by inverting the Fisher information matrix for $(\lambda , \theta )$ given by

5 $$\begin{aligned} I(\lambda , \theta )&=\left[ \begin{array}{cc} I_{11}(\lambda , \theta ) &{} I_{12}(\lambda , \theta ) \\ I_{21}(\lambda , \theta ) &{} I_{22}(\lambda , \theta ) \end{array} \right] \nonumber \\&=\left[ \begin{array}{cc} E\left( -\frac{\partial ^2}{\partial \lambda ^2} \log L(\theta , \lambda )\right) &{} E\left( -\frac{\partial ^2}{\partial \theta \partial \lambda } \log L(\theta , \lambda )\right) \\ E\left( -\frac{\partial ^2}{\partial \lambda \partial \theta } \log L(\theta , \lambda )\right) &{} E\left( -\frac{\partial ^2}{\partial \theta ^2} \log L(\theta , \lambda ) \right) \end{array} \right] . \end{aligned}$$

The negative second derivatives of the log-likelihood function in (5) are given by

$$\begin{aligned}&-\frac{\partial ^2}{\partial \lambda ^2} \log L(\lambda , \theta ) =\frac{k}{\lambda ^2}, \\&-\frac{\partial ^2}{\partial \theta \partial \lambda } \log L(\lambda , \theta ) =- \frac{2e^{-\theta x_{L(k)}}}{1-e^{-2\theta x_{L(k)}}} x_{L(k)},\\&-\frac{\partial ^2}{\partial \theta ^2} \log L(\lambda , \theta ) =\frac{k}{\theta ^2}+\lambda \frac{2e^{-\theta x_{L(k)}}(1+e^{-2\theta x_{L(k)}})}{\left( 1-e^{-2\theta x_{L(k)}}\right) ^2} x_{L(k)}^2 -\sum _{i=1}^{k} \frac{4e^{-2\theta x_{L(i)}}}{\left( 1-e^{-2\theta x_{L(i)}}\right) ^2} x_{L(i)}^2. \end{aligned}$$

Then, with the marginal density function of $X_{L(i)}$,

$$\begin{aligned} f_{X_{L{(i)}}}(x)=&\frac{2 \theta \lambda ^i }{\Gamma (i)} \left[ \log \left( \frac{1+e^{-\theta x}}{1-e^{-\theta x}} \right) \right] ^{i-1} \left( \frac{1-e^{-\theta x}}{1+e^{-\theta x}} \right) ^\lambda \frac{e^{-\theta x}}{1-e^{-2\theta x}}, \quad x>0, \quad i=1, \ldots , k, \end{aligned}$$

and the series expansions (see Jeffrey and Zwillinger 2007)

$$\begin{aligned} \log \left( \frac{1+z}{1-z}\right)&=2 \sum _{j=1}^{\infty } \frac{z^{2j-1}}{2j-1} \quad \mathrm {for} \quad z^2<1, \\ \left[ \log (1 \pm z)\right] ^2&=2 \sum _{j=1}^{\infty } (\mp 1)^{j+1}\frac{z^{j+1}}{j+1} \sum _{i=1}^{j}\frac{1}{i} \quad \mathrm {for} \;\; z^2<1, \\ \log \left( {1+z}\right) \log \left( {1-z}\right)&= -\sum _{j=1}^{\infty } \frac{z^{2j}}{j} \sum _{i=1}^{2j-1}\frac{(-1)^{i+1}}{i} \quad \mathrm {for} \;\; z^2<1, \end{aligned}$$

the Fisher information matrix (5) can be obtained as

6 $$\begin{aligned} I(\lambda , \theta )&=\left[ \begin{array}{ll} k/\lambda ^2 &{} \quad Q_2(\lambda )/\theta \\ Q_2(\lambda )/\theta &{}  \quad Q_1(\lambda )/\theta ^2 \end{array} \right] , \end{aligned}$$

where

$$\begin{aligned}&Q_1(\lambda )=k+\frac{\lambda ^{k+1}[h_5(\lambda )+h_6(\lambda )]}{2}- \sum _{i=1}^k \frac{\lambda ^i[h_7(\lambda )+h_8(\lambda )]}{2}, \\&Q_2(\lambda )=-\lambda ^k h_9(\lambda ), \\&h_5(\lambda )=\sum _{j=1}^{\infty } \frac{1+(-1)^{j+1}}{j+1} \left( \frac{1}{(j-1+\lambda )^k}- \frac{1}{(j+3+\lambda )^k}\right) \sum _{l=1}^{j}\frac{1}{l}, \\&h_6(\lambda )=\sum _{j=1}^{\infty } \frac{1}{j} \left( \frac{1}{(2j-2+\lambda )^k} -\frac{1}{(2j+2+\lambda )^k}\right) \sum _{l=1}^{2j-1}\frac{(-1)^{l+1}}{l}, \\&h_7(\lambda )=\sum _{j=1}^{\infty } \frac{1+(-1)^{j+1}}{j+1} \left( \frac{1}{(j-1+\lambda )^i}+ \frac{1}{(j+3+\lambda )^i}-\frac{2}{(j+1+\lambda )^i}\right) \sum _{l=1}^{j}\frac{1}{l}, \\&h_8(\lambda )=\sum _{j=1}^{\infty } \frac{1}{j} \left( \frac{1}{(2j-2+\lambda )^i}+\frac{1}{(2j+2+\lambda )^i}-\frac{2}{(2j+\lambda )^i}\right) \sum _{l=1}^{2j-1}\frac{(-1)^{l+1}}{l}, \\&h_9(\lambda )=\sum _{j=1}^{\infty } \frac{1}{2j-1}\left( \frac{1}{(2j-2+\lambda )^k}- \frac{1}{(2j+\lambda )^k}\right) . \end{aligned}$$

Therefore, under the suitable regularity conditions (Lehmann and Casella 1998), by the asymptotic normality of the MLE, the approximate $100(1-\alpha )\%$ CIs based on the MLEs can be constructed as

7 $$\begin{aligned} \hat{\theta }\pm z_{\alpha /2} \sqrt{\mathrm {Var} \left( \hat{\theta }\right) } \quad \mathrm {and} \quad \hat{\lambda }\pm z_{\alpha /2} \sqrt{\mathrm {Var} \left( \hat{\lambda }\right) }, \end{aligned}$$

where $z_{\alpha /2}$ denotes the upper $\alpha /2$ point of the standard normal distribution and the variances $\mathrm {Var} \left( \hat{\lambda }\right)$ and $\mathrm {Var} \left( \hat{\theta }\right)$ are the diagonal elements of the asymptotic variance-covariance matrix $I^{-1}\left( \hat{\lambda }, \hat{\theta }\right)$. However these CIs can yield negative lower bounds although both $\lambda$ and $\theta$ are supported by $(0, \infty )$. The next subsection provides estimation methods based on pivotal quantities that can address the disadvantage of approximate CIs (7) and are much easier to calculate than the maximum likelihood method.

### Estimation based on pivotal quantities

Wang et al. (2015) provided some lemmas to construct exact confidence intervals for the family of proportional reversed hazard distributions based on lower record values. Based on their results, this subsection develops estimation methods based on pivotal quantities.

Let

$$\begin{aligned} Z_i&=-\log \left[ F(x_{L(i)}) \right] \nonumber \\&=-\lambda \log \left( \frac{1-e^{-\theta x_{L(i)}}}{1+e^{-\theta x_{L(i)}}} \right) , \quad i=1, \ldots , k. \end{aligned}$$

Then, by Lemma 1 in Wang et al. (2015), iid RVs with the standard exponential distribution can be obtained as

$$\begin{aligned} S_i&=Z_i-Z_{i-1} \nonumber \\&=\lambda \left[ \log \left( \frac{1-e^{-\theta x_{L(i-1)}}}{1+e^{-\theta x_{L(i-1)}}} \right) -\log \left( \frac{1-e^{-\theta x_{L(i)}}}{1+e^{-\theta x_{L(i)}}} \right) \right] , \quad i=1, \ldots , k \, (Z_0 \equiv 0). \end{aligned}$$

From the spacing, a pivotal quantity for a gamma distribution with parameters (*k*, 1) is given by

8 $$\begin{aligned} T_k&=\sum _{i=1}^{k} S_i \nonumber \\&= \lambda h_1(\theta ). \end{aligned}$$

Then, because $h_1(\theta )$ has a gamma distribution with parameters ($k, \lambda$), an unbiased estimator of $\lambda$ is given by

9 $$\begin{aligned} \hat{\lambda }_p(\theta )&=\frac{k-1}{ h_1(\theta ) }, \end{aligned}$$

which has an inverse gamma distribution with parameters $(k, \lambda (k-1))$ and its MSE is given by

$$\begin{aligned} \hbox {MSE} \left( \hat{\lambda }_p (\theta ) \right) =\frac{\lambda ^2}{k-2}, \quad k>2. \end{aligned}$$

Note that since the MLE $\hat{\lambda }(\theta )$ has an inverse gamma distribution with parameters $(k, \lambda k)$ from the fact that $h_1(\theta )$ has a gamma distribution with parameters ($k, \lambda$), its bias and MSE are given respectively by

$$\begin{aligned} \hbox {Bias} \left( \hat{\lambda }(\theta ) \right) =\frac{\lambda }{k-1} \end{aligned}$$

and

$$\begin{aligned} \hbox {MSE} \left( \hat{\lambda }(\theta ) \right)&=\frac{\lambda ^2(k+2)}{(k-2)(k-1)}, \quad k>2. \end{aligned}$$

In addition, since $2T_k$ has a $\chi ^2$ distribution with 2*k* degrees of freedom, for $0<\alpha <1$, we have

$$\begin{aligned} 1-\alpha&=P\left[ \chi ^2_{1-\alpha /2, \, 2k}<2T_k<\chi ^2_{\alpha /2, \, 2k} \right] \\&=P\left[ \frac{\chi ^2_{1-\alpha /2, \, 2k}}{2h_1(\theta )}<\lambda < \frac{\chi ^2_{\alpha /2, \, 2k}}{2h_1(\theta )} \right] , \end{aligned}$$

where $\chi ^2_{\alpha , \, k}$ is the upper $\alpha$ percentile of a $\chi ^2$ distribution with *k* degrees of freedom. Therefore, an exact $100(1-\alpha )\%$ CI for $\lambda$ is given by

10 $$\begin{aligned} \left( \frac{\chi ^2_{1-\alpha /2, \, 2k}}{2h_1(\theta )}, \; \frac{\chi ^2_{\alpha /2, \, 2k}}{2h_1(\theta )} \right) . \end{aligned}$$

Note that the unbiased estimator (9) and the exact $100(1-\alpha )\%$ CI (10) depend on the nuisance parameter $\theta$. Now the method for addressing the nuisance parameter $\theta$ is discussed.

For the pivotal quantity (8), the pivotal quantities

$$\begin{aligned} T_j&=\sum _{i=1}^{j} S_i \nonumber \\&= \lambda \log \left( \frac{1+e^{-\theta x_{L(j)}}}{1-e^{-\theta x_{L(j)}}} \right) , \quad j=1, \ldots , k \end{aligned}$$

can be obtained such that they are independent RVs having a gamma distribution with parameters (*j*, 1). Then, since $2T_j$ are independent RVs having a $\chi ^2$ distribution with 2*j* degree of freedom, the transforms

$$\begin{aligned} U_j&=\left( \frac{T_j}{T_{j+1}} \right) ^j \\&=\left[ \frac{\log \left( \left( 1+e^{-\theta x_{L(j)}} \right) \Big / \left( 1-e^{-\theta x_{L(j)}} \right) \right) }{\log \left( \left( 1+e^{-\theta x_{L(j+1)}} \right) \Big / \left( 1-e^{-\theta x_{L(j+1)}} \right) \right) } \right] ^j, \quad j=1, \ldots , k-1 \end{aligned}$$

are iid RVs with a uniform distribution on (0, 1). Therefore, the pivotal quantity

$$\begin{aligned} W(\theta )&=-2\sum _{j=1}^{k-1} \log U_j \end{aligned}$$

can be obtained such that it has a $\chi ^2$ distribution with $2k-2$ degrees of freedom. Here an estimator of $\theta$, $\hat{\theta }_p$, can be found by solving the equation $W(\theta )/(2k-4)=1$ for $\theta$ because $W(\theta )/(2k-4)$ converges in probability to 1 as $k \rightarrow \infty$. Note that the estimator $\hat{\theta }_p$ is unique because $W(\theta )$ is a strictly increasing function of $\theta$ (see “Appendix”). Then, by substituting the estimator $\hat{\theta }_p$ for $\theta$ in the unbiased estimator (9), the estimator (9) is written as

11 $$\begin{aligned} \hat{\lambda }_p\left( \hat{\theta }_p \right)&=\frac{k-1}{ h_1\left( \hat{\theta }_p \right) }, \end{aligned}$$

which is no longer an unbiased estimator. In addition, an exact $100(1-\alpha )\%$ CI for $\lambda$ can be obtained by using the pivotal quantity $W(\theta )$ when $\theta$ is unknown. The following theorem provides an exact $100(1-\alpha )\%$ CI for $\lambda$ when $\theta$ is unknown.

#### **Theorem 1**

*Let*$\theta ^*$*be the unique solution of*$W(\theta )=W$, *whereW* has a $\chi ^2$*distribution with*$2(k-1)$*degree of freedom. Then, an exact*$100(1-\alpha )\%$*CI for*$\lambda$*based on a generalized pivotal quantity*$W \left( \theta ^* \right)$*is*

12 $$\begin{aligned} \left( W \left( \theta ^* \right) _{1-\alpha /2}, W \left( \theta ^* \right) _{\alpha /2} \right) , \end{aligned}$$

*where*$W \left( \theta ^* \right) _{\alpha }$*is the upper*$\alpha$* percentile of the generalized pivotal quantity*$W \left( \theta ^* \right) = {T_k}/{h_1 \left( \theta ^* \right) }$*obtained using* (8) and $\theta ^*$.

The percentiles of the generalized pivotal quantity $W \left( \theta ^* \right)$ are obtained based on the following algorithm:

Step 1.: Generate *W* from a $\chi ^2$ distribution with $2(k-1)$ degree of freedom and solve the equation $W(\theta )=W$ for $\theta$ to obtain $\theta ^*$.
Step 2.: Generate $2T_k$ from the $\chi ^2$ distribution with 2*k* degree of freedom.
Step 3.: Compute $$W \left( \theta ^* \right) $$.
Step 4.: Repeat $N(\ge 10,000)$ times.

As mentioned before, the Bayesian approach is a good alternative for small sample sizes. The next section provides the Bayesian estimation methods.

## Bayesian estimation

Seo and Kang (2014) assumed independently distributed gamma priors to draw inferences for the EHLD based on lower record values. If there is sufficient information on the prior, then these subjective priors are appropriate. However, it is not easy to obtain such information in practice. Therefore, this section derives a reference prior for $(\lambda , \theta )$ when $\lambda$ is the parameter of interest. In addition, a robust estimation method is developed by constructing a hierarchical model of the subjective marginal prior $\pi (\lambda )$. The procedure is as follows: a subjective marginal prior for $\lambda$ is supposed to estimate the parameter of interest $\lambda$, and then derive a conditional reference prior for $\theta$ for $\lambda$ by considering the scenario proposed in Sun and Berger (1998). Then the joint prior for $(\lambda , \theta )$ is derived. Based on this prior, a robust estimation method is developed.

### Noninformative prior

The Jeffrey and reference priors can be derived based on the Fisher information matrix (6). By definition of Jeffreys (1961), the Jeffrey prior for $(\lambda , \theta )$ is given by

13 $$\begin{aligned} \pi (\lambda , \theta ) = \frac{1}{\theta } \sqrt{\frac{k}{\lambda ^2}Q_1(\lambda )-Q_2^2(\lambda )}, \quad \frac{\left[ \lambda Q_2(\lambda ) \right] ^2}{Q_1(\lambda )}<k. \end{aligned}$$

To derive the reference prior for $(\lambda , \theta )$ when $\lambda$ is the parameter of interest, the algorithm of Beger and Bernardo (1989) is applied as follows.

Let

$$\begin{aligned} \pi (\theta | \lambda )&= \sqrt{I_{22}(\lambda , \theta )} \\&=\frac{1}{\theta } \sqrt{Q_1(\lambda )} \end{aligned}$$

and by choosing a sequence of compact sets $\Omega _i=(d_{1i}, \, d_{2i}) \times (d_{3i}, \, d_{4i})$ for $(\lambda , \theta )$ such that $d_{1i}, d_{3i} \rightarrow 0$, $d_{2i}, d_{4i} \rightarrow \infty$ as $i \rightarrow \infty$, it follows that

$$\begin{aligned} K_{i}^{-1}(\lambda )&=\int _{d_{3i}}^{d_{4i}} \pi (\theta | \lambda ) \; d \theta \\&= \sqrt{Q_1(\lambda )}\left[ \log (d_{4i})-\log (d_{3i}) \right] \end{aligned}$$

and

$$\begin{aligned} p_i(\theta | \lambda )&=K_{i}(\lambda ) \pi (\theta | \lambda ) \mathbf{{1}}_{(d_{3i}, \, d_{4i})}(\theta ) \nonumber \\&=\frac{1}{\theta \left[ \log (d_{4i})-\log (d_{3i})\right] } \mathbf{{1}}_{(d_{3i}, \, d_{4i})}(\theta ), \end{aligned}$$

where $\mathbf{{1}}_{\Omega }$ denotes the indicator function on $\Omega$. In addition, the marginal reference prior for $\lambda$ is given by

$$\begin{aligned} \pi _i(\lambda )&= \exp \left[ \frac{1}{2} \int _{d_{3i}}^{d_{4i}} p_i(\theta | \lambda ) \log \left( \frac{|I(\lambda , \theta )|}{I_{22}(\lambda , \theta )}\right) d \theta \right] \\&=\sqrt{\frac{k}{\lambda ^2}-\frac{Q_2^2(\lambda )}{Q_1(\lambda )}}, \end{aligned}$$

where |*I*| denotes the determinant of matrix *I*. Then the reference prior is obtained as

14 $$\begin{aligned} \pi _{R}(\lambda , \theta )&=\lim _{i \rightarrow \infty }\left[ \frac{K_{i}(\lambda ) \pi _i(\lambda )}{K_{i}(\lambda _0) \pi _i(\lambda _0)} \right] \pi (\theta | \lambda ) \nonumber \\&\propto \frac{1}{\theta }\sqrt{\frac{k}{\lambda ^2}-\frac{Q_2^2(\lambda )}{Q_1(\lambda )}}, \quad \frac{\left[ \lambda Q_2(\lambda ) \right] ^2}{Q_1(\lambda )}<k, \end{aligned}$$

where $\lambda _0$ is any fixed point.

Note that because the Jeffreys prior (13) and the reference prior (14) have constraints on the parameter $\lambda$ to obtain positive square roots, these noninformative priors are ineffective in objective Bayesian analysis. Therefore, the next subsection develops a robust estimation method by constructing a hierarchical structure of the parameter of interest $\lambda$.

### Robust estimation

Suppose that the marginal prior is a gamma prior with the PDF

15 $$\begin{aligned} \pi (\lambda |\alpha , \beta ) = \frac{\beta ^{\alpha }}{\Gamma (\alpha )} \lambda ^{\alpha -1}e^{-\beta \lambda }, \quad \alpha , \beta >0, \end{aligned}$$

which is a conjugate prior when the nuisance parameter $\theta$ is known. In addition, based on Theorem 2 of Sun and Berger (1998), a conditional reference prior $\pi (\theta |\lambda )$ is given by

$$\begin{aligned} \pi (\theta |\lambda )&\propto \sqrt{I_{22}(\lambda , \theta )}\\&=\frac{Q_1(\lambda )}{\theta ^2}\\&\propto \frac{1}{\theta }. \end{aligned}$$

Then the joint prior density function for $(\lambda , \theta )$ is given by

16 $$\begin{aligned} \pi (\lambda , \theta |\alpha , \beta )= \frac{\beta ^{\alpha }}{\Gamma (\alpha )} \theta ^{-1} \lambda ^{\alpha -1}e^{-\beta \lambda }. \end{aligned}$$

From the likelihood function (1) and the joint prior density function (16), the joint posterior density function for $(\lambda , \theta )$ is given by

17 $$\begin{aligned} \pi (\lambda , \theta | \alpha , \beta , \mathbf{x})&= \frac{L(\lambda , \theta ) \pi (\lambda , \theta | \alpha , \beta )}{\int _{\theta }\int _{\lambda }L(\lambda , \theta ) \pi (\lambda , \theta | \alpha , \beta ) d\lambda d\theta } \nonumber \\&\propto \left[ \prod _{i=1}^{k} \frac{e^{-\theta x_{L(i)}}}{1-e^{-2\theta x_{L(i)}}} \right] \theta ^{k-1} \lambda ^{k+\alpha -1}e^{-\lambda (\beta +h_1(\theta ))}. \end{aligned}$$

The marginal posterior density function for $\theta$ is obtained by integrating the joint posterior density function (17) as

18 $$\begin{aligned} \pi (\theta | \alpha , \beta , \mathbf{x})&= \int _{\lambda }\pi (\lambda , \theta | \alpha , \beta , \mathbf{x}) d \lambda \nonumber \\&\propto \frac{\theta ^{k-1}}{\left[ \beta +h_1(\theta ) \right] ^{k+\alpha }} \prod _{i=1}^{k} \frac{e^{-\theta x_{L(i)}}}{1-e^{-2\theta x_{L(i)}}}. \end{aligned}$$

Here the nuisance parameter $\theta$ can be simply estimated by maximizing the marginal posterior density function (18), which is denoted by $\hat{\theta }_{MAP}$. Then because the marginal posterior density function for $\lambda$ is given by

19 $$\begin{aligned} \pi (\lambda |\alpha , \beta , \mathbf{x})&= \int _{\theta } \pi (\lambda |\alpha , \beta , \theta , \mathbf{x}) \pi (\theta |\alpha , \beta ,\mathbf{x}) d\theta , \end{aligned}$$

where $\pi (\lambda |\alpha , \beta , \theta , \mathbf{x})$ is the conditional posterior density function for $\lambda$, with $\theta$ substituted by $\hat{\theta }_{MAP}$ in $\pi (\lambda |\alpha , \beta , \theta , \mathbf{x})$, the marginal posterior density function (19) can be approximated as

$$\begin{aligned} \pi (\lambda |\alpha , \beta , \mathbf{x})&\approx \frac{\left[ \beta + h_1\left( \hat{\theta }_{MAP} \right) \right] ^{k+\alpha }}{\Gamma (k+\alpha )} \lambda ^{k+\alpha -1} e^{-\lambda \left[ \beta + h_1\left( \hat{\theta }_{MAP} \right) \right] }, \end{aligned}$$

which is the PDF of the gamma distribution with parameters $\left( k+\alpha , \beta +h_1\left( \hat{\theta }_{MAP} \right) \right)$.

Note that if $\alpha \rightarrow 0$ and $\beta \rightarrow 0$, then the posterior mean $\hat{\lambda }_B(\theta )$ is the same as the MLE $\hat{\lambda }(\theta )$ and the posterior mode $\hat{\lambda }_{MAP}(\theta )$ is the same as the unbiased estimator $\hat{\lambda }_p(\theta )$ when the nuisance parameter $\theta$ is known. However since the marginal distributions for $\lambda$ and $\theta$ depend on hyperparameters $\alpha$ and $\beta$, if there is no sufficient information on the prior, then values of the hyperparameters cannot be determined. Now, a method for addressing these hyperparameters for robust estimation results is developed.

Let $\pi (\alpha , \beta )$ be a joint prior for the hyperparameters $\alpha$ and $\beta$. Then

20 $$\begin{aligned} \pi (\lambda , \alpha , \beta ) =\pi (\lambda |\alpha , \beta ) \pi (\alpha , \beta ). \end{aligned}$$

Based on Han (1997), values of $\alpha$ and $\beta$ should be determined such that the marginal prior $\pi (\lambda |\alpha , \beta )$ is a decreasing function of $\lambda$. For $0<\alpha \le 1$ and $\beta >0$, the condition is satisfied. However, $\beta$ is restricted to the support (0, *c*) because prior distributions with very thin tails may sensitive. In addition, only the case of $\alpha =1$ is considered for simplicity. Then the marginal prior $\pi (\lambda |\alpha , \beta )$ can be written as

$$\begin{aligned} \pi (\lambda |\beta ) = \beta e^{-\beta \lambda }, \quad 0<\beta <c. \end{aligned}$$

For robust inferences, assign a uniform distribution on (0, *c*) as a prior distribution of $\beta$. Then the hierarchical prior is given by

21 $$\begin{aligned} \pi (\lambda , \theta )&= \pi (\theta |\lambda ) \int _{0}^c \pi (\lambda |\beta ) \pi (\beta ) d\beta \nonumber \\&=\frac{1}{c\theta } \int _{0}^c \beta e^{-\beta \lambda }d\beta \nonumber \\&=\frac{1}{c\theta \lambda ^2} \left[ 1-(1+c\lambda )e^{-c\lambda } \right] . \end{aligned}$$

Therefore, the joint posterior density function for $(\lambda , \theta )$ under the hierarchical prior (21) is given by

22 $$\begin{aligned} \pi (\lambda , \theta | \mathbf{x})&= \frac{L(\lambda , \theta ) \pi (\lambda , \theta )}{\int _{\theta }\int _{\lambda }L(\lambda , \theta ) \pi (\lambda , \theta ) d\lambda d\theta } \nonumber \\&\propto \left[ \prod _{i=1}^{k} \frac{e^{-\theta x_{L(i)}}}{1-e^{-2\theta x_{L(i)}}} \right] \theta ^{k-1} \lambda ^{k-2}\left[ 1-(1+c\lambda )e^{-c\lambda } \right] e^{-\lambda h_1(\theta )}. \end{aligned}$$

The marginal posterior density function for $\theta$ is obtained by integrating the joint posterior density function (22) as

23 $$\begin{aligned} \pi (\theta | \mathbf{x})&= \int _{\lambda }\pi (\lambda , \theta | \mathbf{x}) d \lambda \nonumber \\&\propto \frac{\theta ^{k-1}}{h_2\left( \theta \right) } \prod _{i=1}^{k} \frac{e^{-\theta x_{L(i)}}}{1-e^{-2\theta x_{L(i)}}}, \end{aligned}$$

where

$$\begin{aligned} h_2\left( \theta \right) =\frac{1}{\left[ h_1\left( \theta \right) \right] ^{k-1}} - \frac{ck+h_1\left( \theta \right) }{\left[ c+h_1\left( \theta \right) \right] ^{k}}. \end{aligned}$$

Here the nuisance parameter $\theta$ can be estimated by maximizing the marginal posterior density function (23), and it is denoted by $\hat{\theta }_{HMAP}$. The marginal posterior density function for $\lambda$ is provided in the following theorem.

#### **Theorem 2**

*The marginal posterior density function for the parameter of interest*, $\lambda$, *is approximated as*

24 $$\begin{aligned} \pi (\lambda | \mathbf{x})&\approx \frac{\lambda ^{k-2}\left[ 1-(1+c\lambda )e^{-c\lambda } \right] e^{-\lambda h_1\left( \hat{\theta }_{HMAP} \right) }}{\Gamma (k-1) h_2\left( \hat{\theta }_{HMAP} \right) }. \end{aligned}$$

#### *Proof*

The marginal posterior density function for $\lambda$ is given by

25 $$\begin{aligned} \pi (\lambda | \mathbf{x})&= \int _{\theta } \pi (\lambda |\theta , \mathbf{x}) \pi (\theta |\mathbf{x}) d\theta , \end{aligned}$$

where $\pi (\lambda |\theta , \mathbf{x})$ is the conditional posterior density function for $\lambda$ for $\theta$. With $\theta$ substituted by $\hat{\theta }_{HMAP}$ in $\pi (\lambda |\theta , \mathbf{x})$, the marginal posterior density function (25) can be approximated as $\pi \left( \lambda |\hat{\theta }_{HMAP}, \mathbf{x} \right)$, and this completes the proof. $\square$

#### **Corollary 1**

*From the marginal posterior density function* (24), *the corresponding posterior mean is*

$$\begin{aligned} \hat{\lambda }_{HB} \left( \hat{\theta }_{MAP} \right)&=\int _{\lambda } \pi (\lambda | \mathbf{x}) d\lambda \\&=\frac{(k-1)h_3\left( \hat{\theta }_{HMAP} \right) }{h_2\left( \hat{\theta }_{HMAP} \right) }, \end{aligned}$$

*where*

$$\begin{aligned} h_3\left( \hat{\theta }_{HMAP} \right) =\frac{1}{\left[ h_1\left( \hat{\theta }_{HMAP} \right) \right] ^{k}} - \frac{c(k+1)+h_1\left( \hat{\theta }_{HMAP} \right) }{\left[ c+h_1\left( \hat{\theta }_{HMAP} \right) \right] ^{k+1}}. \end{aligned}$$

*Therefore if*$c \rightarrow \infty$, *then the posterior mean*$\hat{\lambda }_{HB} \left( \theta \right)$*is the same as the unbiased estimator*$\hat{\lambda }_p(\theta )$*when the nuisance parameter*$\theta$ is known.

## Application

This section assesses the proposed methods and verifies them using real data.

### A simulation study

For a simulation study, the proposed estimators are compared in terms of their MSEs and biases over 10,000 replications. Among the estimators $\hat{\phi }_i(i=1,2)$ of the unknown parameter $\phi$, if the MSE of $\hat{\phi }_1$ is smaller the that of $\hat{\phi }_2$ for any value of $\phi$, then $\hat{\phi }_1$ is preferable and can be said to be more efficient than the other. For the bias, the same argument is made. The Bayes estimator $\hat{\lambda }_B \left( \hat{\theta }_{MAP} \right)$ is computed based on a vague prior with the hyperparameters $\alpha =\beta =0.01$. To examine the robustness of the hierarchical prior (21), the Bayes estimator $\hat{\lambda }_{HB} \left( \hat{\theta }_{HMAP} \right)$ is computed for different $c=5, 100, 500$. Lower record values are generated from the standard EHLD with $\lambda =2(2)8$. In addition, coverage probabilities (CPs) of CIs based on the MLE $\hat{\lambda }\left( \hat{\theta }\right)$ and the generalized pivotal quantity $W(\theta ^*)$ are reported at the 0.95 confidence level based on 10,000 simulations. These values are given in Table 1. Because the parameter of interest is $\lambda$, no results for the nuisance parameter $\theta$ are reported here.

**Table 1:** MSEs(biases) and $95\,\%$ CIs for $\lambda$

$\lambda$	*k*	MSE(bias)			*c*	MSE(bias)	CI based on	
		$\hat{\lambda }$	$\hat{\lambda }_p \left( \hat{\theta }_p \right)$	$\hat{\lambda }_B \left( \hat{\theta }_{MAP} \right)$		$\hat{\lambda }_{HB} \left( \hat{\theta }_{HMAP} \right)$	$\hat{\lambda }$	$W \left( \theta ^* \right)$
2	10	0.357 (0.239)	0.175 (0.005)	0.260 (0.115)	5	0.170 (−0.003)	0.977	0.954
					100	0.173 (−0.004)		
					500	0.173 (−0.004)		
	12	0.230 (0.180)	0.135 (0.003)	0.179 (0.086)	5	0.132 (−0.002)	0.972	0.951
					100	0.135 (−0.002)		
					500	0.135 (−0.002)		
	14	0.151 (0.139)	0.100 (−0.003)	0.123 (0.065)	5	0.099 (−0.002)	0.971	0.949
					100	0.100 (−0.002)		
					500	0.100 (−0.002)		
	16	0.121 (0.119)	0.085 (−0.003)	0.101 (0.056)	5	0.084 (−0.001)	0.970	0.950
					100	0.085 (−0.001)		
					500	0.085 (−0.001)		
4	10	0.771 (0.330)	0.270 (0.020)	0.427 (0.178)	5	0.228 (−0.004)	0.972	0.954
					100	0.229 (−0.005)		
					500	0.229 (−0.005)		
	12	0.406 (0.230)	0.189 (0.012)	0.266 (0.126)	5	0.172 (−0.005)	0.968	0.951
					100	0.172 (−0.005)		
					500	0.172 (−0.005)		
	14	0.208 (0.167)	0.120 (0.001)	0.157 (0.089)	5	0.115 (−0.003)	0.968	0.950
					100	0.115 (−0.003)		
					500	0.115 (−0.003)		
	16	0.151 (0.136)	0.096 (0.000)	0.119 (0.072)	5	0.094 (−0.002)	0.966	0.949
					100	0.094 (−0.002)		
					500	0.094 (−0.002)		
6	10	1.923 (0.454)	0.468 (0.046)	0.624 (0.227)	5	0.294 (0.004)	0.975	0.954
					100	0.294 (0.004)		
					500	0.294 (0.004)		
	12	0.783 (0.298)	0.289 (0.027)	0.383 (0.159)	5	0.224 (0.002)	0.968	0.951
					100	0.224 (0.002)		
					500	0.224 (0.002)		
	14	0.306 (0.204)	0.153 (0.008)	0.208 (0.110)	5	0.141 (−0.001)	0.967	0.950
					100	0.141 (−0.001)		
					500	0.141 (−0.001)		
	16	0.195 (0.159)	0.113 (0.003)	0.146 (0.086)	5	0.108 (−0.001)	0.966	0.950
					100	0.108 (−0.001)		
					500	0.108 (−0.001)		
8	10	5.376 (0.604)	0.871 (0.079)	0.769 (0.264)	5	0.355 (0.009)	0.975	0.952
					100	0.355 (0.009)		
					500	0.355 (0.009)		
	12	1.522 (0.377)	0.452 (0.047)	0.488 (0.188)	5	0.279 (0.006)	0.967	0.951
					100	0.279 (0.006)		
					500	0.279 (0.006)		
	14	0.458 (0.247)	0.202 (0.018)	0.268 (0.130)	5	0.171 (−0.003)	0.964	0.950
					100	0.171 (−0.003)		
					500	0.171 (−0.003)		
	16	0.254 (0.186)	0.134 (0.009)	0.176 (0.099)	5	0.124 (−0.003)	0.962	0.950
					100	0.124 (−0.003)		
					500	0.124 (−0.003)		

Table 1 shows that $\hat{\lambda }_{HB} \left( \hat{\theta }_{HMAP} \right)$ is generally more efficient than other estimators in terms of the MSE and bias. In addition, it is quite robust to the choice *c*. CPs of CIs based on the MLE exceed corresponding nominal levels, whereas those of CIs based on the generalized pivotal quantity are well matched to their corresponding nominal levels.

### Real data

This subsection analyzes real data considered in Seo and Kang (2014), which represent the amount of annual rainfall (in inches) recorded at the Los Angeles Civic Center from 1877 to 2012. From the data, 10 lower record values are observed as

$$\begin{aligned} 21.26, \quad 11.35, \quad 10.40, \quad 9.21, \quad 6.73, \quad 5.59, \quad 5.58, \quad 4.85, \quad 4.42, \quad 3.21. \end{aligned}$$

**Fig. 1:**
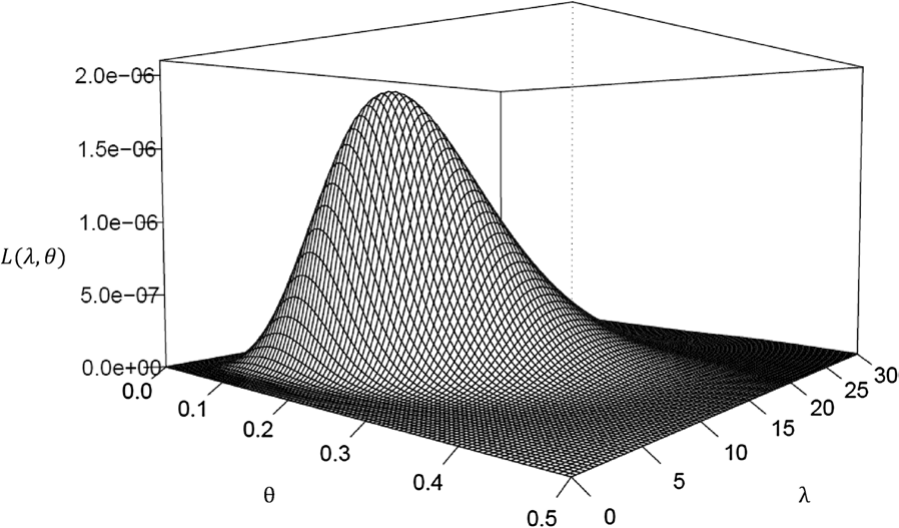
Likelihood function of $\lambda$ and $\theta$

**Fig. 2:**
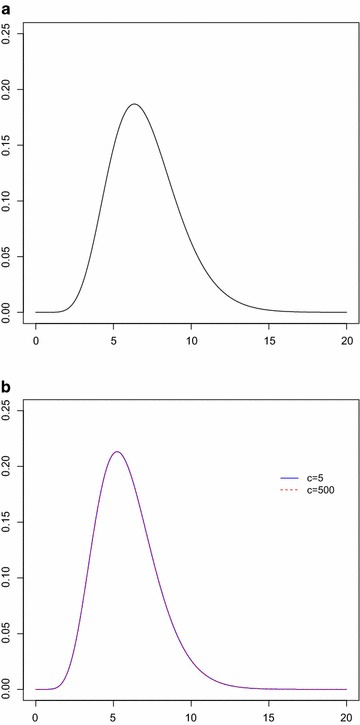
**a** Marginal posterior density function $\pi (\lambda |\alpha , \beta , \mathbf{{x}})$ and **b** Marginal posterior density function $\pi (\lambda |\mathbf{{x}})$

Seo and Kang (2014) showed that the EHLD provides a good fit to both whole data and observed lower record values. The record values are used to obtain estimates discussed in previous sections. For Bayesian inferences, hyperparameters $\alpha =\beta =0.01$ are set because no information on the prior is given. For the proposed hierarchical model, consider different $c=5, 100, 500$. Estimates of the nuisance parameter $\theta$ are reported in Table 2, and estimates of the parameter of interest $\lambda$, and corresponding $95\,\%$ intervals are reported in Table 3. Figure 1 plots the likelihood function of unknown parameters ($\lambda , \theta$) and Fig. 2 plots the marginal posterior density functions for $\lambda$ derived in “Frequentist estimation” section.

**Table 2:** Proposed estimates of $\theta$

$\hat{\theta }$	$\hat{\theta }_p$	$\hat{\theta }_{MAP}$	$\hat{\theta }_{HMAP}$		
			$c=5$	$c=100$	$c=500$
0.184	0.138	0.156	0.137	0.137	0.137

**Table 3:** Proposed estimates of $\lambda$

	$\hat{\lambda }$	$\hat{\lambda }_p\left( \hat{\theta }_p \right)$	$\hat{\lambda }_B \left( \hat{\theta }_{MAP} \right)$	$\hat{\lambda }_{HB}\left( \hat{\theta }_{HMAP} \right)$		
				$c=5$	$c=100$	$c=500$
Estimates	7.996	5.897	7.051	5.891	5.890	5.890
Intervals	(1.433, 14.560)	(2.739, 15.661)	(3.055, 11.530)	(2.428, 9.889)	(2.428, 9.889)	(2.428, 9.889)
Lengths	13.127	12.922	8.475	7.461	7.461	7.461

Tables 2 and 3 show that the Bayes estimates under the hierarchical prior (21) have the same values for different values of *c*. The credible intervals have shorter lengths than CIs. In addition, it is observed that the MLEs $\hat{\lambda }$ and $\hat{\theta }$ are exist and unique from Fig. 1. Figure 2 shows that the marginal posterior density function has the same shape for different values of *c*. These results indicate that the Bayesian approach based on the hierarchical prior (21) produces robust results and is superior to non-Bayesian approach in terms of the interval length.

## Conclusion

This paper proposes more efficient methods for estimating shape and scale parameters of the EHLD based on record values from Bayesian and non-Bayesian perspectives. The results verify that the method based on the pivotal quantity is superior to the maximum likelihood method in terms of the MSE, bias, and CP of the CI based on Monte Carlo simulations and that it is more computationally convenient. In addition, it is noted that the Bayes estimator of $\lambda$ under the hierarchical prior (21) is a generalized version of the unbiased estimator of $\lambda$ when the nuisance parameter $\theta$ is known. Through Monte Carlo simulations and real data analysis, it was verified that not only the Bayesian estimation under the hierarchical prior (21) are more efficient than the estimation based on the pivotal quantity in terms of the MSE and bias, but estimation results under the hierarchical prior (21) are robust to *c*. Therefore, the proposed robust Bayesian estimation method should be used if there is no sufficient prior information.

## Appendix

This appendix shows that the proposed estimator $\hat{\theta }_p$ is unique.

Let

$$\begin{aligned} G_j(\theta )= \left( \frac{1-e^{-\theta x_{L(j)}}}{1+e^{-\theta x_{L(j)}}} \right) , \quad j=1, \ldots , k, \end{aligned}$$

which is the same as the CDF of the HLD with the parameter $\theta$. Then the pivotal quantity $W(\theta )$ can be written as

$$\begin{aligned} W(\theta )=2\sum _{j=1}^{k-1}\left[ \log \left( -\log G_k(\theta ) \right) - \log \left( -\log G_j (\theta )\right) \right] \end{aligned}$$

and by differentiating it for $\theta$, the following derivative is obtained:

26 $$\begin{aligned} \frac{\partial }{\partial \theta }W(\theta )= 2\sum _{j=1}^{k-1} \left( \frac{g_k(\theta ) }{G_k(\theta ) \log G_k (\theta )} -\frac{g_j(\theta ) }{G_j(\theta ) \log G_j (\theta ) } \right) , \end{aligned}$$

where

$$\begin{aligned} g_j\left( \theta \right)&=\frac{\partial }{\partial \theta } G_j(\theta ) \\&= \frac{2 x_{L(k)} e^{-\theta x_{L(j)}}}{\left[ 1+e^{-\theta x_{L(j)}} \right] ^2}, \quad j=1, \ldots , k. \end{aligned}$$

To show that the term $g_j(\theta ) / \left( G_j(\theta ) \log G_j (\theta ) \right)$ is decreasing in $x_{L(j)}$, decompose it as

27 $$\begin{aligned} \frac{g_j(\theta ) }{G_j(\theta ) \log G_j (\theta )} = \frac{1}{\left( 1+e^{-\theta x_{L(j)}}\right) \log G_j(\theta )} \frac{x_{L(j)} e^{-\theta x_{L(j)}}}{1-e^{-\theta x_{L(j)}}}. \end{aligned}$$

In the right side of (27), it can be easily shown that the derivative of the first term is negative and the derivative of the second term is given by

28 $$\begin{aligned} \frac{\partial }{\partial x_{L(j)}}\frac{x_{L(j)} e^{-\theta x_{L(j)}}}{1-e^{-\theta x_{L(j)}}} =\frac{e^{-\theta x_{L(j)}}\left( 1-e^{-\theta x_{L(j)}}-\theta x_{L(j)} \right) }{\left( 1-e^{-\theta x_{L(j)}} \right) ^2}. \end{aligned}$$

Define $\varphi \left( x_{L(j)} \right) =1-e^{-\theta x_{L(j)}}-\theta x_{L(j)}$. Note that $\varphi \left( x_{L(j)} \right)$ has supremum zero and it is a strictly decreasing function of $x_{L(j)}$ because

$$\begin{aligned} \frac{\partial }{\partial x_{L(j)}}\varphi \left( x_{L(j)} \right)&= \theta \left( e^{-\theta x_{L(j)}}-1 \right) \\&<0. \end{aligned}$$

That is, the derivative (28) is negative. Then, because (27) is a strictly decreasing function of $x_{L(j)}$, the function (26) is positive, and the pivotal quantity $W(\theta )$ is a strictly increasing function of $\theta$. Therefore the equation $W(\theta )/(2k-4)=1$ has a unique solution.
